# IsomiRage: From Functional Classification to Differential Expression of miRNA Isoforms

**DOI:** 10.3389/fbioe.2014.00038

**Published:** 2014-09-29

**Authors:** Heiko Muller, Matteo Jacopo Marzi, Francesco Nicassio

**Affiliations:** ^1^Center for Genomic Science of IIT@SEMM, Istituto Italiano di Tecnologia (IIT), Milan, Italy

**Keywords:** miRNA, isomiRs, next-generation sequencing, pipeline, alignment, cancer

## Abstract

As more small RNA sequencing libraries are becoming available, it clearly emerges that microRNAs (miRNAs) are highly heterogeneous both in length and sequence. In comparison to canonical miRNAs, miRNA isoforms (termed as “isomiRs”) might exhibit different biological properties, such as a different target repertoire, or enhanced/reduced stability. Nonetheless, this layer of information has remained largely unexplored due to the scarcity of small RNA NGS-datasets and the absence of proper analytical tools. Here, we present a workflow for the characterization and analysis of miRNAs and their variants in next-generation sequencing datasets. IsomiRs can originate from an alternative dicing event (“templated” forms) or from the addition of nucleotides through an enzymatic activity or target-dependent mechanisms (“non-templated” forms). Our pipeline allows distinguishing canonical miRNAs from templated and non-templated isomiRs by alignment to a custom database, which comprises all possible 3′-, 5′-, and trimmed variants. Functionally equivalent isomiRs can be grouped together according to the type of modification (e.g., uridylation, adenylation, trimming …) to assess which miRNAs are more intensively modified in a given biological context. When applied to the analysis of primary epithelial breast cancer cells, our methodology provided a 40% increase in the number of detected miRNA species and allowed to easily identify and classify more than 1000 variants. Most modifications were compatible with templated IsomiRs, as a consequence of imprecise Drosha or Dicer cleavage. However, some non-templated variants were consistently found either in the normal or in the cancer cells, with the 3′-end adenylation and uridylation as the most frequent events, suggesting that miRNA post-transcriptional modification frequently occurs. In conclusion, our analytical tool permits the deconvolution of miRNA heterogeneity and could be used to explore the functional role of miRNA isoforms.

## Introduction

microRNAs (miRNAs), a small (18–25 nt long), evolutionarily conserved class of non-coding RNAs, are important regulators of transcriptional programs by silencing the expression of a multitude of target mRNAs at a post-transcriptional level (Bartel, 2009). The biogenesis of miRNAs typically requires a nuclear cleavage of the primary transcript by the Drosha/DGCR8 complex and a cytoplasmic cleavage of the hairpin-folded precursor miRNA (pre-miRNA) by Dicer [reviewed in Krol et al. (2010)]. The product of this cleavage is usually a mature 21/22 bp miRNA duplex, which is loaded onto the RNA-induced silencing complex (RISC) to function in the miRNA silencing mechanism (Gregory et al., 2005). Only one strand is retained in the RISC, usually the one with unstable base-pairing at its 5′-end, and it mediates target repression through base complementarity between the miRNA “seed region” (nucleotides 2–7) and the miRNA responsive elements (MRE), mostly located at the 3′ untranslated region (3′UTR) of target genes (Bartel, 2009).

Generally, each mature miRNA is annotated as a unique mature sequence (the reference or canonical miRNA sequence) and could derive from either the 5′ or 3′ arm of the same pre-miRNA hairpin (termed as -5p or -3p, respectively). However, the recent advent of next-generation sequencing has clearly shown that mature miRNAs can be present in several sequence variants or isoforms, named “isomiRs” [reviewed in Neilsen et al. (2012)]. Initially, isomiRs were considered as sequencing artifacts, but a growing body of evidence revealed that isomiRs are actual miRNA variants that can exert a biological activity. For instance, isomiRs are found associated with Argonaute proteins in the RISC complex as canonical miRNAs and could exert silencing of a specific target in *in vitro* luciferase assays (Lee et al., 2010; Cloonan et al., 2011). The generation of isomiRs is heterogeneous. In fact, they can originate from imprecise cleavage by Drosha or Dicer (the so-called “templated isomiRs”), which generates variants that show perfect sequence complementarity to their pre-miRNA. Alternatively, isomiRs could be generated by post-transcriptional modifications due to enzymatic activity, which could either add or remove specific nucleotides to miRNA ends. These miRNA variants are known as “non-templated isomiRs,” with sequence imperfectly matching their pre-miRNA. Typically, non-templated modifications occur at the 3′end, while 5′end isomiRs are rare (Newman et al., 2011; Wyman et al., 2011). This is likely due to fact that a 5′-end modification (templated or non-templated) actually modifies the target repertoire of the miRNA, which is dictated by the “seed” region [nucleotide 2–7 (Bartel, 2009)]. The expression profiles of miRNA variants are dynamic, with differences across tissues or cell lines (Landgraf et al., 2007). Nonetheless, the functional significance of isomiRs has remained elusive due to the limited number of tools available to specifically monitor their levels in sequencing experiments [e.g., isomiRex (Sablok et al., 2013), miRNA-MATE (Cloonan et al., 2011), miRAnalyzer (Hackenberg et al., 2009)]. In sporadic cases, it was shown that isomiRs could alter the target specificity (Azuma-Mukai et al., 2008), the efficiency of Ago loading (Burroughs et al., 2010), or the half-life (Katoh et al., 2009) of the cognate miRNA. Regardless of their biological activity, many isomiRs are highly expressed, even more than the corresponding canonical miRNAs. Thus, their annotation is particularly relevant in order to properly analyze expression profiles and eventually identify contexts where miRNA isoforms could be functional.

Here, we describe a pipeline that allows the identification and analysis of all miRNA variants (canonical miRNAs and “templated” or “non-templated” isomiRs) from small RNA sequencing experiments (Illumina). These variants could be grouped according to the site (5′-end or 3′-end) or the type of modification (trimming, adenylation, uridylation …) to assess the extent of miRNA modifications in a given biological context. As a proof-of-principle analysis, we applied our methodology to analyze miRNAs and isomiRs expression in human samples (i.e., primary normal and breast cancer cells), revealing that miRNA modifications frequently occur and may significantly affect global miRNA expression and regulation.

## Materials and Methods

### Small RNA samples: Cell culture, RNA isolation, and small RNA sequencing

The samples described in this work were prepared from a triple-negative breast cancer primary culture and its normal counterpart as described in Pece et al. (2004). The epithelial origin of the cultures was confirmed by immunofluorescence with an anti-Pan cytokeratin antibody (Sigma-Aldrich). All tissues were collected at the European Institute of Oncology via standardized operative procedures approved by the Institutional Ethical Board, and informed consent was obtained for all tissue specimens. Total RNA, including small species, was isolated through the miRNeasy mini kit (Qiagen). One microgram of total RNA was used to prepare Small RNA libraries following the Illumina TruSeq™ Small RNA Sample Preparation Guide, as by manufacturers’ instructions. The libraries were sequenced at 50 bp single-read mode and 80 million read depth on an Illumina HiSeq 2000 platform. All the relevant steps of the *IsomiRage* analysis workflow are fully described in the text. Sequencing results are listed in Table S1 in Supplementary Material. Raw data together with detailed description of the procedures are available in GEO database (GSE21090).

### Quantitative real time PCR

RT-qPCR reactions were performed in triplicate using the miScript RT system in conjunction with miScript primer assays (Qiagen), as by manufacturers’ protocol. One microgram of total RNA was used to prepare cDNA. U6b was used as housekeeping.

### Statistical analysis

Microsoft Excel was used to generate bar graphs. Bivariate analyses, pie-chart, and statistics (Fisher’s test, Student’s *t*-test) were performed using JMP 10 (SAS) software.

### IsomiRage JAVA tool

*IsomiRage* is a standalone desktop application written in the Java programing language. It was developed using NetBeans 7.3.1 Integrated Development Environment software. IsomiRage requires Java 1.6 to run and has been tested on Window 7 and MacOS 10 operating systems. The *IsoMirRagetool* (updated to the latest miRbase release, miRbase 21) is available at http://cru.genomics.iit.it/Isomirage/.

## Results

**Figure 1:**
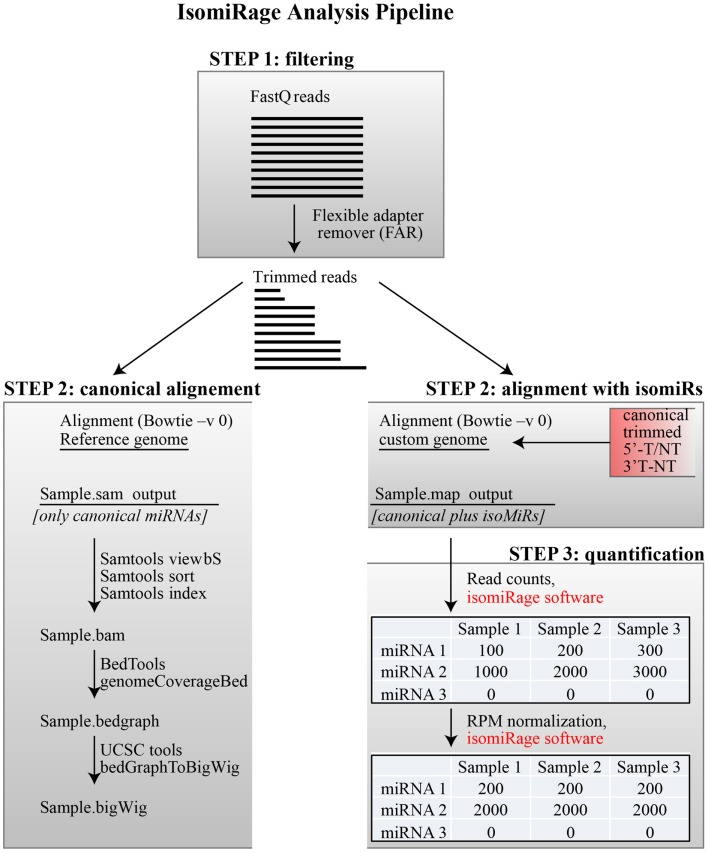
**IsomiRage pipeline**. A schematic representation of the miRNA analysis workflow is shown, distinguishing the three main steps described in the text. Alignment of trimmed FastQ reads (Step 2) is carried out either using a reference genome for the production of browser viewable tracks or to custom sequence libraries for quantitative analyses of miRNA and their isoforms. The quantification of mapped reads in this latter case is performed through an *ad hoc* java application called *IsomiRage*, which also performs the read-per-million normalization and provides, for each miRNA, the different kinds of modifications found.

We present a pipeline, named as “*IsomiRage*,” for profiling the miRNAs/isomiRs and corresponding differential expression patterns using Illumina next-generation sequencing datasets of small RNA. We discuss the application of *IsomiRage* to the analysis of small RNA sequencing data obtained by matched normal and tumor primary breast cell culture with the Illumina Hiseq 2000 sequencing system. The *IsomiRage* workflow has three main steps summarized in Figure 1: filtering of reads, alignment on a custom genome, and quantification and normalization of IsomiRs.

### Step 1 – filtering of reads

**Figure 2:**
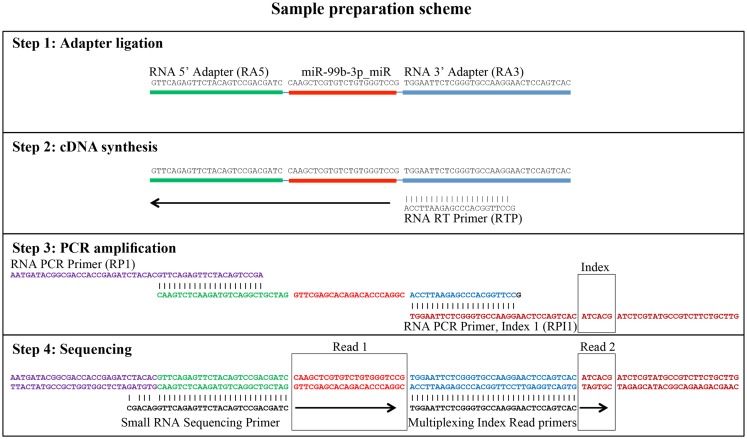
**Generation of small RNA sequencing libraries**. The major steps from sample preparation to sequencing for small RNA molecules according to the Illumina protocol (Illumina TruSeq™ Small RNA Sample Preparation) are shown. The steps include adapter ligation, first strand cDNA synthesis, PCR-amplification, and sequencing. The regions of homology of the adapters involved are shown. The sequence of miR-99b-3p was chosen as an example. Read 1 indicates the sequencing cycles producing miRNA related sequence data. Read 2 indicates the production of index reads used for de-multiplexing of samples. Note that the miRNA related reads may contain adapter sequence at their 3′ end, which needs to be removed prior to downstream analyses.

**Figure 3:**
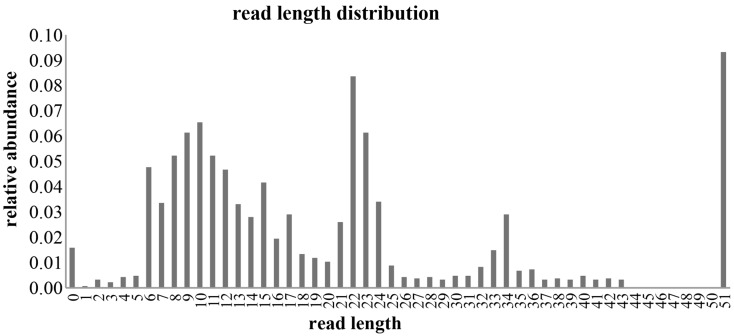
**Distribution of filtered reads**. The distribution of read-lengths after adapter removal is shown. The distribution is multi-modal with peaks at 0, 10, 22, 34, and 51 bases. Mode 22 represents the miRNA related reads that are considered in this study.

Small RNA libraries are routinely prepared following the Illumina TruSeq™ Small RNA Sample Preparation guide, shown in Figure 2. Different biological samples are marked with specific 6 bp sequencing indices to allow multiplexing. According to our experience, up to 12 different small RNA libraries can be pooled in a single sequencing lane to obtain up to 18 million filtered reads from each library. Sequencing is performed in single-read mode with read length of 50 bp. De-multiplexing is carried out using CASAVA software to produce reads in FastQ format for each biological sample. Adapters used during library preparation are removed using The Flexible Adapter Remover software^1^ (FAR version 2.15). As shown in Figure 2, adapter sequence may be found only at the 3′-end of the reads and corresponds to adapters RA3 and RPI, which have identical 5′-ends. FAR typically produces collections of reads whose lengths are distributed in a multi-modal distribution as shown in Figure 3. The largest mode is located at length 22, which corresponds to miRNAs. Minor modes can be observed at length 10, 34, 0, and 51. The first (length 10) marks the peak of range of read-lengths between 6 and 17 bases, which likely represent break-down products. The reads of the 34-bases mode show homology to tRNAs. The two last modes are at length 0 and length 51. The former corresponds to PCR fragments not containing any successfully cloned RNA molecules while the latter represents PCR fragments with RNA molecules longer than 50 bases or where adapter removal has failed for other reasons. Adapter removal may fail when the fraction of the adapter represented in the read is too short for being recognized as an adapter-derived sequence or when the adapter sequence contains errors. It is worth noting that adapter removal is essential for successful alignment of small RNA reads to a reference genome.

### Step 2 – alignment

Filtered reads are aligned to a custom genome that includes the sequences of all canonical mature miRNAs^2^ (2578 human and 1975 mouse miRNAs, according to the release 20 of miRBASE) and their related isomiRs (5′-end, 3′-end and trimmed variants, shown in Table S1 in Supplementary Material). The variant sequences were generated including all the possible combinations of one, two, or three bases extending the 5′- or the 3′-end of known miRNA sequences plus the sequences obtained by trimming canonical miRNA from their 3′-end down to a length of 18 bp (reads below 18 bp were not considered since the alignment would be unreliable). Each isomiR is associated to a series of feature, including the corresponding canonical miRNA, the site of modification (5′-end or 3′-end), the type of modification (trimming; addition of one, two, or three nucleotides; type of nucleotide/s added), and the origin of the isomiR (“templated” or “non-templated”). The latter definition was based on the alignment of each isomiR to the sequence of the pre-miRNA. A perfect pairing with the pre-miRNA sequence is associated to “templated” variants, while non-perfect pairing is associated to “non-templated” variants. It is worth mentioning that there is still a probability that a “templated” base variation might be a *de novo* modification, rather than an imprecise cleavage by Drosha or Dicer. Alignment is performed with the Bowtie ultrafast short-read aligner in the -v 0 alignment mode, which specifies that no mismatches are allowed (Figure 1). Only the best alignment is reported for each read. The Bowtie output is stored in .map textual format and supplied as input to a custom software (i.e., *IsomiRage*) for downstream analyses. Filtered reads could also be aligned to the reference genome. In this case, the Bowtie output is stored in SAM format (Li et al., 2009) and contains data only for canonical miRNAs. The Bowtie output is processed further to produce browser viewable bam and bigwig files. These files can be used for qualitative analyses.

### Step 3 – quantification and normalization

To estimate the expression level of a given miRNA or IsomiR, the number of perfectly aligned Illumina reads are counted by *IsomiRage* JAVA tool (available at http://cru.genomics.iit.it/Isomirage/). The software reads the .map Bowtie output file and ensures that the read aligns perfectly to the chosen reference. Of note, this approach works only if the Bowtie output contains one and only one reported alignment for each Illumina read. This is achieved using the Bowtie -v 0 switch together with the best switch. The output is a table that lists the number of reads for each isoform in each biological condition (see Figure 1). We routinely obtain three to five million reads for each biological condition. To enable quantitative comparisons between samples, the read numbers must be normalized for sequencing depth. This step is carried out by standard read-per-million (RPM) normalization, providing a table of RPM-normalized read counts that can be used for comparisons of fold changes and other downstream analyses.

### Application of the pipeline: Small RNA sequencing of normal and cancer breast cells

#### Alignment and sequencing output

**Figure 4:**
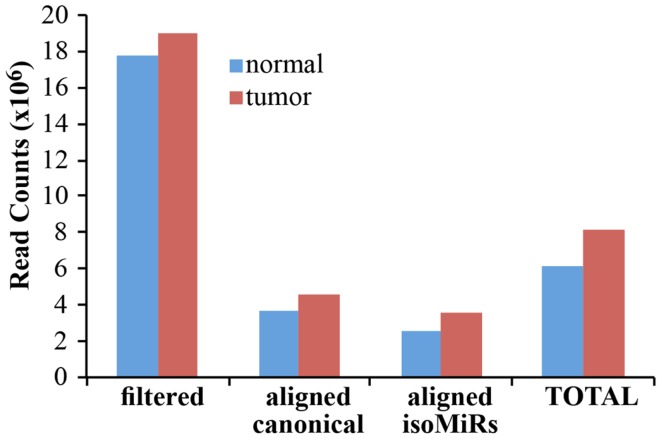
**Alignment of filtered reads**. The cumulative amount of reads, after adapter removal, is shown for the two samples analyzed. “Filtered” reads refer to the sum of all the species reported in Figure 3. Of note, the alignment on the custom genome required a read to be at least 18 nucleotides in length. “Total” is the sum of canonical miRNAs and all their isoforms.

As a proof-of principle analysis, we applied our methodology to analyze miRNAs and isomiRs expression in real samples. We sequenced small RNAs from 1 μg of total RNA obtained from a matched normal/tumor primary culture pair of breast epithelium. We obtained about 18 million filtered reads for each sample, of which about 7 million were aligned to the custom genome (Table S1 in Supplementary Material; Figure 4). Of note, approximately 4 million reads could be mapped to canonical miRNAs, claiming that with our pipeline the sequencing output could be improved almost twofold (Figure 4). The improvement in the sequencing output has been similarly observed across multiple experiments and samples (not shown), regardless of the number of multiplexed samples (from 2 to 12). Considering the data from the normal and the tumor sample as a whole, we obtained at least 1 read for 1228 different miRNA species, of which 318 present with >100 counts in at least 1 sample (Table S1 in Supplementary Material). Of note, without considering IsomiRs, we would have identified only 876 miRNAs, 219 having >100 reads. Thus, our pipeline considerably expands the number of detected species and increases the number of mapped reads almost twofold.

#### Differential expression analysis

**Figure 5:**
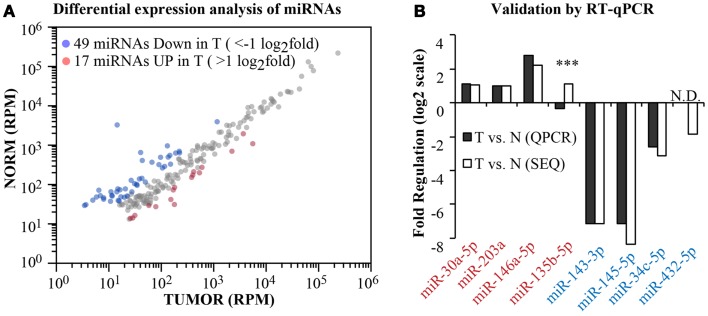
**Differential expression analysis**. **(A)** A scatter plot shows the expression level of canonical miRNAs in the normal and tumor samples. Data have been normalized in reads-per-million (RPM) and shown in log_10_ scale. Blue and red dots identify those miRNAs with >1 log_2_ fold regulation between the two samples. **(B)** Comparison of the fold change as measured by RNA-seq or by RT-qPCR (miScript assay, Qiagen) for eight randomly selected miRNAs (four upregulated and four downregulated). Three asterisks mark non-concordant results. N.D., no PCR data.

Having obtained the aligned data, we moved on to analyze the differential expression of canonical miRNAs in the tumor compared to the normal sample (Figure 5). To calculate fold changes, data where normalized to total read counts (RPM). We selected those miRNA robustly expressed (>100 reads) and identified 66 miRNAs differentially regulated (|*x*| > 1 log_2_ fold, Figure 5A). Among these, we selected randomly four upregulated and four downregulated miRNAs and measured their expression level by RT-qPCR (Figure 5B). For one miRNA, namely, miR-432-5p, RT-qPCR was not sensitive enough to detect the miRNA either in the normal or in the tumor sample (N.D., no data). Nonetheless, as shown in Figure 5B, 6/7 miRNAs were found concordantly regulated by the two methodologies (RNA-seq vs. RT-qPCR), both in qualitative and quantitative terms. Thus, our pipeline produces precise measurements of mature miRNA levels, with an 86% validation rate by an independent method.

#### Expression of isomiRs: the case of miR-92a

**Figure 6:**
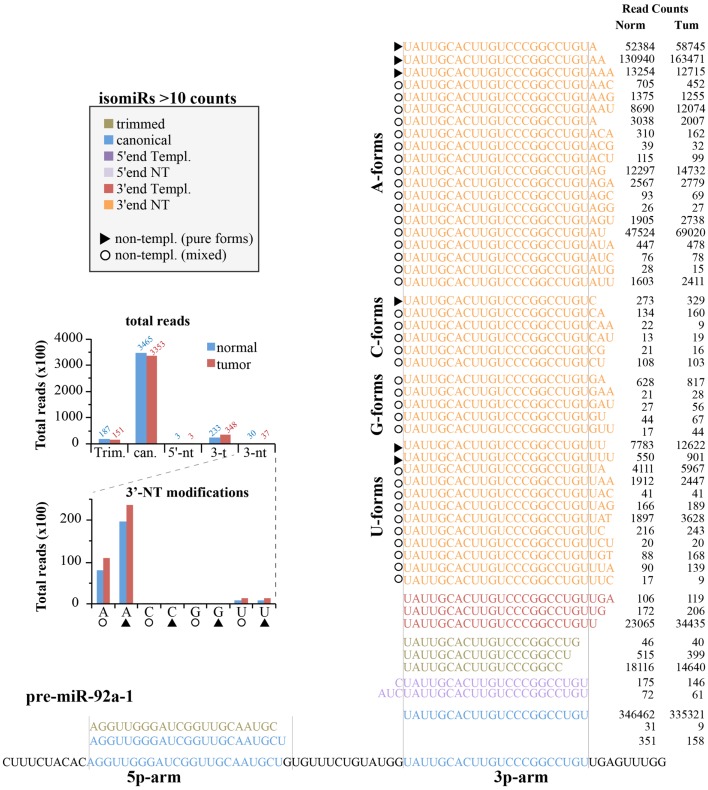
**IsomiRs from miR-92a-1 locus**. Figure summarizes all the isoforms identified (>10 read counts) for the hsa-miR-92a-1 locus according to the *IsomiRage* pipeline. Isoforms are aligned over the precursor miRNA, shown at the bottom. Regions corresponding to the canonical hsa-mir-92a-5p and hsa-miR-92a-3p are highlighted in blue. Isoforms are grouped according to their type of modification (5′-end, 3′-end, trimmed forms, and canonical sequences). Isoforms perfectly pairing with the precursor miRNA (shown at the bottom) are designated as “templated” (templ.), otherwise we refer to them as “non-templated” (nt). Non-templated modifications at the 3′-end are further grouped according to the first non-templated nucleotide (A-forms, C-forms, G-forms, U-forms). As explained in the text, we could distinguish “mixed” forms (identified by circles), with different type of added nucleotides from “pure” forms (identified by triangles), which bears the same kind of nucleotide, likely as consequence of the same enzymatic activity. A bar graph summarizing the quantification of miR-92a isoforms is shown in the insert.

We next analyzed the expression of canonical miRNAs together with their isomiRs. Figure 6 shows the locus of human pre-miR-92a-1 with the 5p- and 3p-arms and their related mature miRNA species found in the small RNA sequencing experiments. As expected, most of the variants (shown those with >10 counts) come from the 3p-arm, which is the usual processed arm (see miRbase 20 as reference), including 5′-end, 3′-end variants, and trimmed forms. For this miRNA, the canonical form is the prevalent one (>80% of all reads are the canonical hsa-miR-92a-3p; see Figure 6), followed by the trimmed and the 3′-end templated modifications. The 5′-end variants are poorly represented. There are a huge number of 3′end non-templated modifications, some with a robust level of expression (hundreds or even thousands of read counts, Figure 6). This very heterogeneous class could be grouped based on the type of the first nucleotide added to the mature miRNA, which should correspond to a different enzymatic activity (termed as A-, G-, C-, U-forms). To this regard, it is possible to distinguish “pure” forms (the same nucleotide added one, two, or three times; marked with a triangle in Figure 6) from “mixed” forms (with different nucleotides; marked with a circle in Figure 6). The nucleotide distribution appears very uneven, with the A-forms extremely abundant (pure and mixed equally distributed) followed by the U-forms. Modifications with the C or G bases are extremely rare.

#### Expression of isomiRs at genome-wide level

**Figure 7:**
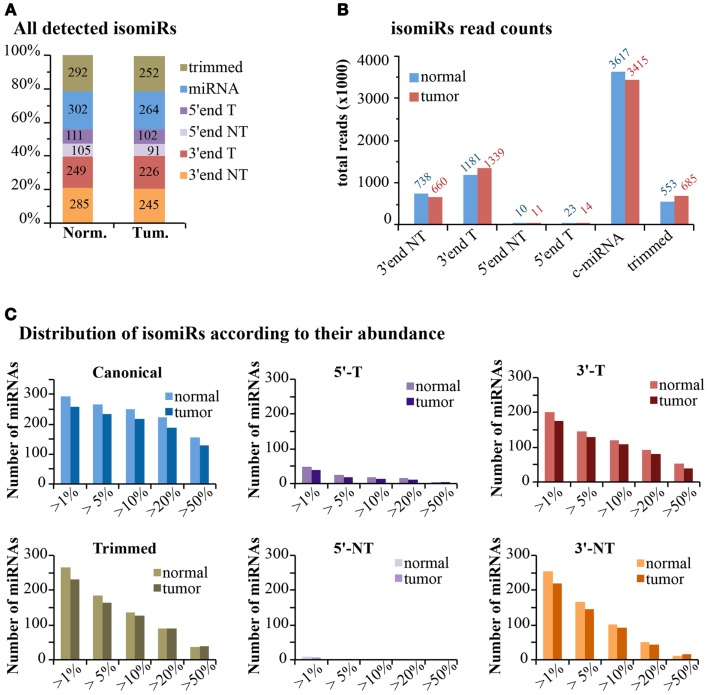
**IsomiRs distribution at genome-wide level**. **(A)** The bar graph shows the percentage of detected IsomiRs (>1 read counts) in the normal and tumor samples, divided in classes, as in Figure 6 (T, templated; NT, non-templated). The absolute number of species is also reported within the bar. **(B)** Bar graph shows the expression levels (total reads) of the each class. **(C)** Those miRNAs robustly detected (total read count for all isoforms >100 reads) were selected. Within each miRNA species, we calculated whether the selected isoform type contributes for at least a given percentage over the total reads of each miRNA.

If we consider the expression of isomiRs at a global scale, we observed that about one-third of all detected species (>1 read count) are composed of canonical miRNAs, while the others come from miRNA variants, mostly at the 3′-end (Figure 7A). This uneven distribution is much more evident when considering read counts, with canonical miRNAs accounting for around 60% of all reads, followed by 3′-end modifications (30%) and 3′trimmed forms (10%) (Figure 7B). The 5′-end modifications only represented 0.4–0.5% of reads. Overall, templated modifications were roughly two-times more expressed than non-templated modifications. We observed little or no differences between the normal and the tumor sample, suggesting that, if any, the dynamic regulation of miRNA modifications is limited to specific isoforms (Figure 7B). Next, we analyzed the impact of variants on the total expression level of each miRNA (Table S1 in Supplementary Material; Figure 7C). Only miRNAs with >100 counts (canonical plus isomiRs) were considered. As expected, the canonical miRNA was the prevalent form (>50% of reads) in more than half of cases. Trimmed forms are well represented and constitute more than 20% of total reads for approximately 100 miRNAs (Figure 7C). Their distribution is similar to the one of 3′-end templated modifications. Indeed, trimmed variants can originate equally from active 3′ shortening (exonucleolytic cleavage) or alternative dicing during miRNA biogenesis. Conversely, 5′-end modifications (templated or non-templated) encompass a minority fraction for each and every miRNA (Figure 7C). Surprisingly, 3′-end non-templated variants, which are unambiguously a product of a post-biogenetic activity, constitute more than 10% of total reads for approximately 100 miRNAs (more than 20% for about 50 miRNAs; Figure 7C). No major differences were observed at a global level in the tumor sample compared to the normal. In fact, only 16 of the 258 (6.2%) miRNAs commonly expressed in the 2 samples showed a variation >10% in 3′ non-templated isomiRs, and 37/258 (14.3%) a variation >5% (Table S1 in Supplementary Material). These data, although coming from just two samples, confirm that the dynamicity of regulation of isomiRs is limited to selected species rather than a global effect.

#### Non-templated 3 ′ end modifications

**Figure 8:**
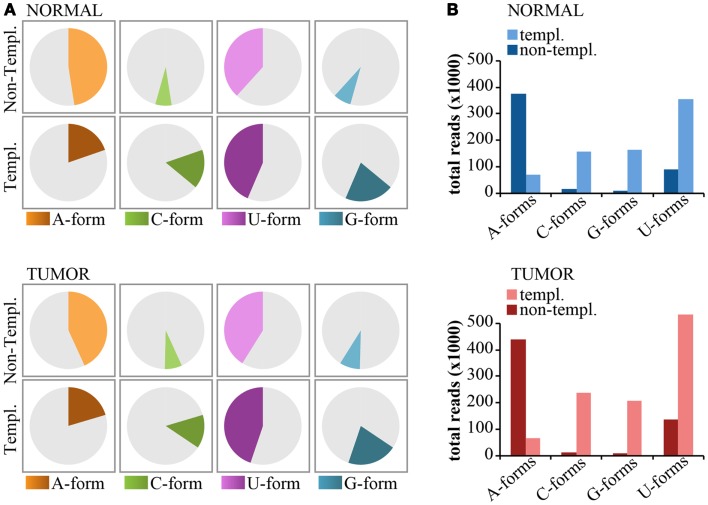
**Nucleotide distribution of 3′-end non-templated modifications**. **(A)** Pie charts show the frequency of 3′ modifications, distinguished according the nucleotide type (A, G, C, U) subdivided in templated or non-templated 3′-end isomiRs. **(B)** The bar graphs show the expression levels (total read counts) of the same species as in **(A)**.

Non-templated 3′-end isomiRs could originate from the activity of nucleotidyl-transferases (Neilsen et al., 2012). These enzymes usually catalyze the addition of uridyl and adenyl nucleotides at the 3′-end of miRNAs. Thus, we expect that uridylation and adenylation should be the prevalent modifications. As shown previously for miR-92a (see Figure 6), we classified 3′-end non-templated (3′-NT) isomiRs into “Iso-groups” (A-forms, C-forms, G-forms, U-forms) according to the nucleotide added at the 3′ end. To be rigorous in our definition, we focused only on the “pure” forms (those with the same nucleotide, e.g., A-forms include only -A, -AA, and -AAA modifications). As shown in Figure 8, adenylation was the most common modification (approximately 50% of the 3′NT modifications are A-forms, Figure 8A) and encompassed most of the reads (Figure 8B) followed by uridylation (50% of the 3′NT modifications and 20% of the reads; Figures 8A,B). Conversely, C- and G-forms accounted only for <5% of the 3′ non-templated modifications (Figures 8A,B). The frequency of adenylation was much higher than expected (*p* < 0.0001 Fisher’s test), even when compared to the frequency of the last nucleotide of the 3′-end templated forms. A very similar trend has been observed in the tumor and in the normal sample (Figures 8A,B).

## Discussion

In the last few years, it became clear that the miRNome is far more complex than previously thought (Lee et al., 2010). The reference sequences reported in miRBase (canonical miRNAs) are usually the prevalent ones, but contemplating the alternative variants, called isomiRs, is crucial to completely understand the complexity of miRNA transcriptome. Here, we described a streamlined pipeline (termed *IsomiRage*) to identify and analyze miRNA isoforms from next-generation sequencing data. The pipeline has been developed for the analysis of data coming from Illumina sequencing, but could be adapted to all the other sequencing methodologies. When applied to real samples (i.e., primary breast normal and cancer cells) *IsomiRage* almost doubled the number of aligned reads and considerably increased the number of detected miRNA species (approximately 40% more species), thus, revealing additional information “hidden” in sequencing datasets.

The identification of isomiRs is based on the alignment to a custom genome, which includes all the possible 3′- end, 5′-end, and trimmed variants for all annotated miRNAs (according to the latest miRBase release). By this approach, the pipeline is able to identify also the non-templated modifications, which are not completely matching with the pre-miRNA molecules and, therefore, missed by standard alignment procedures (that are based on perfect sequence complementarity of miRNAs to the genome or to the pre-miRNA sequence). In line with previous reports (Burroughs et al., 2010; Newman et al., 2011), a huge number of 3′-end non-templated modifications could be detected (>1 read count), several with robust expression (>100 read counts) and contributing to approximately 10–20% of the total reads of a given miRNAs. In extreme cases (11 miRNAs), the 3′-end non-templated isoform was the prevalent one. For instance, miRNAs such as miR-148b-3p, miR-152-3p, or miR-23b-3p displayed highly expressed (>1000 reads) 3′-end non-templated variants. If non-templated forms were not considered, these miRNAs might have been classified as poorly or not expressed. Given the high potential of miRNAs as molecular markers, useful in clinical studies [e.g., circulating miRNA and tumor diagnosis, reviewed in Kosaka et al. (2010)], it will be extremely relevant to consider the expression of both canonical and miRNA variants in these studies, thus, selecting the most expressed (or the most informative) variants as molecular markers.

One possible disadvantage of our approach is that we miss isomiRs that present simultaneously 5′- and 3′-end modifications or polymorphic isomiRs, which harbor substitutions in the internal nucleotide sequence due to genetic differences or epigenetic variations (i.e., editing). Since 5′-end modifications are rarely found, we could speculate that the frequency of concomitant 5′- and 3′-modifications is likely negligible. Similarly, internal variations are very sporadic, with A–I editing being the prevalent type of event and usually limited to specific miRNAs (Kawahara et al., 2007). Indeed, it is worth mentioning that it is always possible to update the custom genome, adding any other classes of modification to extend the coverage of the *IsomiRage* pipeline.

microRNA modifications are extremely heterogeneous and even a single miRNA can display a great number of similar variants (such as has-miR-92a-1, which expressed 43 different non-templated 3′-end isomiRs). Therefore, we propose grouping together functionally equivalent forms to analyze the distribution of non-templated variations at a global scale or at a miRNA-specific level. In the *IsomiRage* workflow, isomiRs are classified according to the site (5′-, 3′-end, or trimming), the origin of modification (templated or non-templated), and the nucleotide of modification (A, G, C, U). Since non-templated modification are believed to occur enzymatically through the activity of nucleotidyl-transferases (Neilsen et al., 2012), we preferred to distinguish those isoforms that bear the same type of added nucleotide (“pure” forms, likely derived from the same enzymatic activity) from those with different nucleotides (“mixed” forms, grouped on the basis of the first non-templated nucleotide).

At a global level, we found that adenylation was by far the most abundant and frequent non-templated modification, followed by uridylation (uridine is also the most frequent last nucleotide of any miRNA), in agreement with previous reports (Burroughs et al., 2010; Newman et al., 2011; Westholm et al., 2012). In plants and lower organisms, these modifications are linked to stabilization or destabilization of miRNAs, respectively (Ramachandran and Chen, 2008; Lu et al., 2009). In mammals, the functions of miRNA post-transcriptional modifications are largely unexplored, likely due to the lack of specific analytical tools. However, they could similarly have important regulatory functions, as shown for the adenylation-mediated stabilization of the liver specific miR-122 (Katoh et al., 2009). In our analysis, which was limited to just one matched tumor vs. normal sample, we did not score a global difference in the extent and the type of non-templated modification. However, when focusing on individual miRNAs, a few of them showed >5% fluctuation in the frequency of adenylated or uridylated forms in the comparison.

One relevant question is why cells have so many miRNA isoforms? As previously mentioned, most of isomiRs are templated variants, originated from imprecise processing of precursor molecules either at 5′- or at 3′-end by the processing enzymes, DGRC8 and Dicer1 (Ameres and Zamore, 2013). These variants are effectively loaded on AGO complexes and, thus, could function as canonical miRNAs (Ebhardt et al., 2009; Cloonan et al., 2011). We can just speculate on the potential usefulness of this “imprecise” machinery. One possibility is that the presence of multiple slightly different variants on the miRISC could improve miRNA functions by increasing the “on-target” to “off-target” ratio (Cloonan et al., 2011). Alternatively, variants could provide opportunity for the evolution of new miRNAs, with similar (3′-end) or different (5′-end) set of targets. For example, a change in 5′ usage might be subsequently fixed by gene duplication and by changes in the precursors miRNA transcript that affects the processing, favoring the so-called “IsomiR switching” (Wheeler et al., 2009; Tan et al., 2014).

In conclusion, using our methodology, it is possible to extend the analysis of small RNA sequencing datasets to reveal a large amount of information that lies unexplored and investigate miRNA post-transcriptional modifications. If applied to large sequencing datasets this approach could uncover the role of isomiRs in the regulation of miRNA expression and function in specific physiological and pathological contexts.

## Author Contributions

Heiko Muller, Matteo Jacopo Marzi, and Francesco Nicassio planned the pipeline. Heiko Muller developed the pipeline for data filtering and alignment together with the *IsomiRage* JAVA tool. Matteo Jacopo Marzi generated the custom genomes and together with Francesco Nicassio performed the analyses reported in the manuscript. Heiko Muller, Matteo Jacopo Marzi, and Francesco Nicassio wrote the manuscript.

## Conflict of Interest Statement

The authors declare that the research was conducted in the absence of any commercial or financial relationships that could be construed as a potential conflict of interest.

## Supplementary Material

The Supplementary Material for this article can be found online at http://www.frontiersin.org/Journal/10.3389/fbioe.2014.00038/abstract

## Footnotes

^1^https://wiki.gacrc.uga.edu/wiki/FAR

^2^http://www.mirbase.org
